# Different Repair Techniques with Excellent Outcomes in Simultaneous Bilateral Patellar Tendon Rupture Following Trampoline Injury: A Case Report

**DOI:** 10.5704/MOJ.2511.017

**Published:** 2025-11

**Authors:** T Jegathesan, HTM Neo

**Affiliations:** 1 Department of Orthopaedic Surgery, Tan Tock Seng Hospital, Singapore; 2 Department of Radiology, National University Hospital, Singapore

**Keywords:** bilateral, patella, tendon, rupture, trampoline

## Abstract

Bilateral patella tendon (BPT) rupture is a rare cause of extensor mechanism failure. It is extremely rare when a young patient without any systemic disease or risk factors, presents with bilateral patella tendon rupture simultaneously. We describe a case of a young lady without predisposing risk factors who sustained a BPT rupture following trauma from jumping on a trampoline. She was admitted inpatient and evaluated with imaging, before eventually undergoing surgical repair of bilateral knee extensor mechanism. Different surgical techniques were employed in both knees with equally excellent outcomes.

## Introduction

Bilateral patella tendon (BPT) rupture is a rare cause of extensor mechanism failure. Many of these patients have predisposing factors such as systemic disease, connective tissue diseases or steroid use that increases their risk. In a cross-sectional study on 229 trampoline-related knee injuries, patella tendon ruptures comprised of 5.4% of such injuries in skeletally mature patients^1^.

We present a case of a young lady who sustained a BPT rupture following jumping on a trampoline without predisposing risk factors. This case draws focus on the possibility of bilateral patella rupture in healthy young patients and describes different surgical techniques that can be used with augmentation to improve the robustness of the repair with excellent outcomes.

## Case Report

Our patient is a 19-year-old Chinese female who is an active cheerleader. She was on the trampoline and somersaulted in mid-air with her bilateral hips and knees in extreme flexion. Upon landing on her feet, her bilateral knees where in a slightly flexed posture and she felt immediate excruciating pain. She was unable to stand up independently. She was brought to the emergency department via ambulance.

**Fig. 1:**
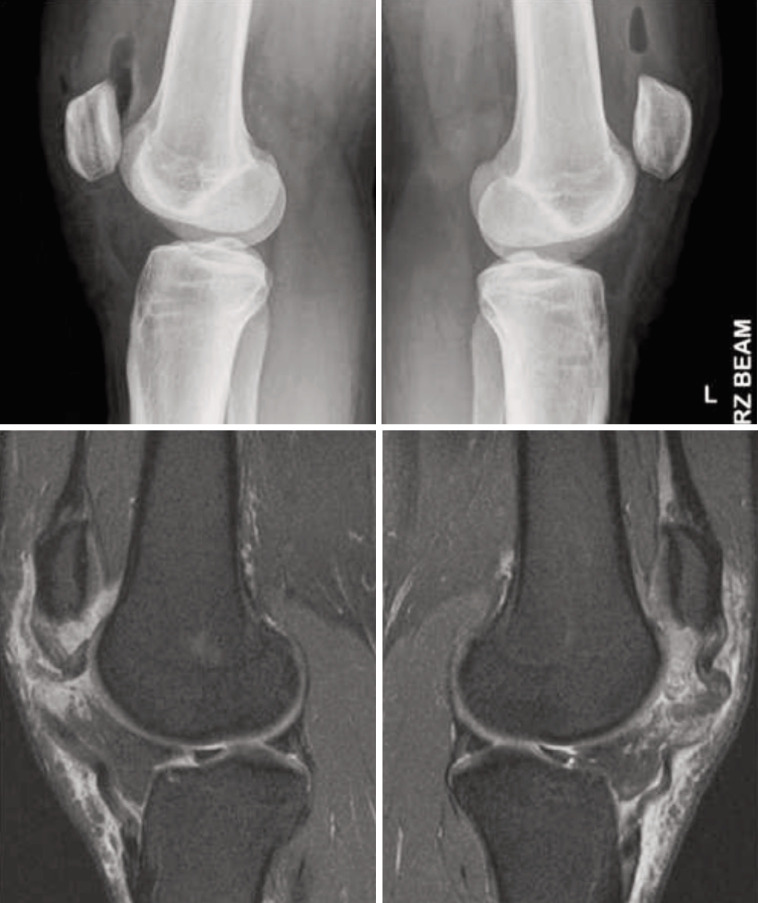
Bilateral lateral radiographs of knees showing patella alta and MRI scans of complete rupture of patella tendons.

She had no past medical history, no family history of connective tissue disorders, nor previous prolonged antibiotic or steroid usage and had engaged in sporting activities without difficulties throughout her teenage years. Physical examination of her bilateral knees showed effusions, with both patellae proximally migrated. There was significant infrapatellar tenderness, loss of active knee extension and a palpable gap noted in both patella tendons’ substance. Radiographs of her knees revealed bilateral high-riding patella or patella alta. MRI scans showed complete rupture of bilateral proximal patellar tendons (Fig. 1).

**Fig. 2:**
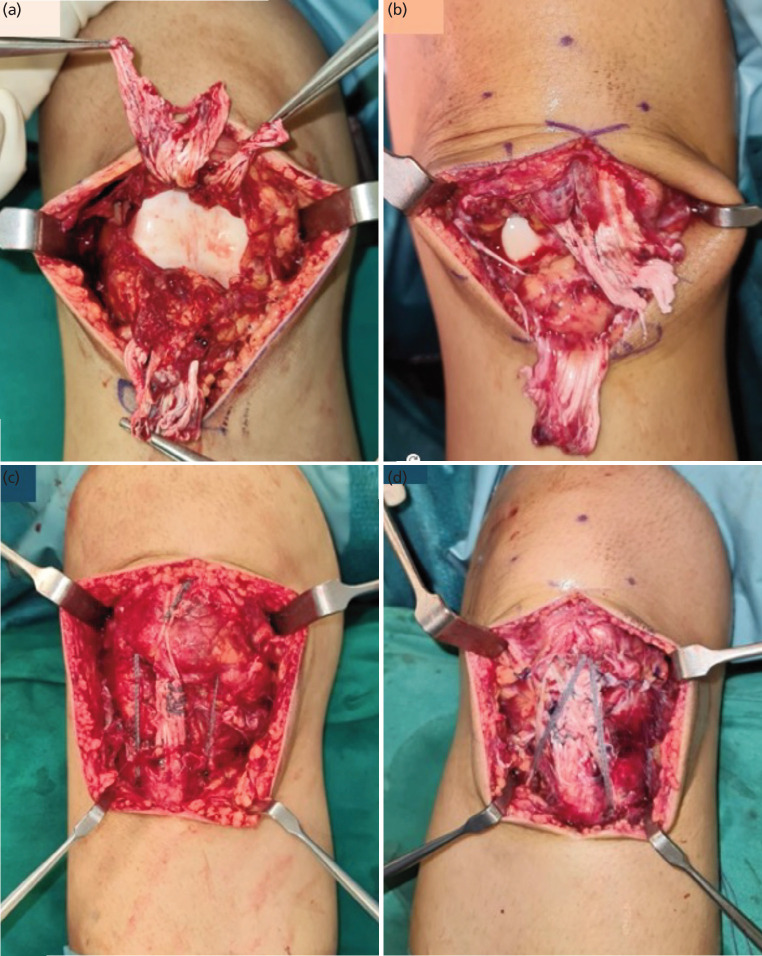
Clinical intra-operative photographs of ruptured patella tendons of the (a) right knee and (b) left knee, and (c) repaired patella tendons of the right knee and (d) left knee.

The patient underwent BPT rupture repair with internal brace. Intra-operatively, there were tears found of both the medial and lateral retinaculum of both knees. It was noticed the ruptured tendon widths were narrow, with patella tendon thickness being thin as well, which may have contributed to this injury (Fig. 2).

**Fig. 3:**
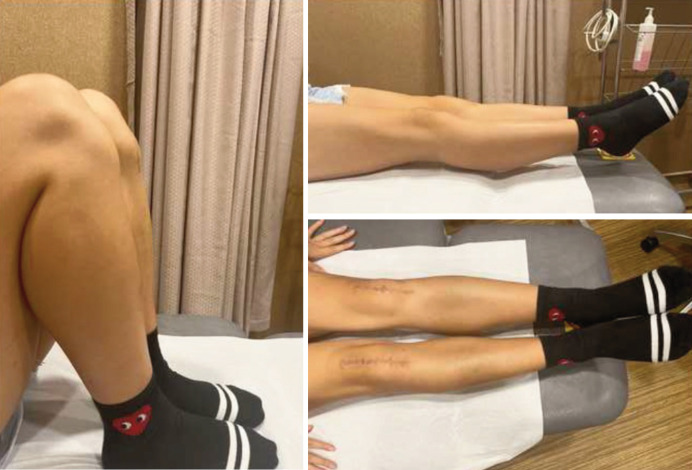
Clinical photographs at 12 months post-operatively showing good knee flexion and extension with corresponding surgical scars.

The right knee had a complete tendon rupture through the proximal fifth of the patella tendon. The unhealthy proximal stump was excised and a transosseous repair of the distal stump was performed. The distal patella tendon stump was tagged with suture tapes in Krakow locking fashion. Three drill holes were placed through the patella, and the suture tapes were passed through the holes and secured over the superior pole of the patella. This transosseous repair was augmented with a “box” internal brace construct, with tapes from anchors inserted at the inferior pole of the patella, before anchoring distally with further anchors to the medial and lateral aspects of the tibial tuberosity with the knee in 30° of flexion (Fig. 3). The retinaculum was then repaired before closure.

The left knee had a complete tendon rupture through the mid-portion of the patella tendon. Quality of the tendon was amenable to direct repair and was performed with four limbs of suture tape in Krakow locking fashion. The retinaculum was then repaired first before augmentation, reinforcing with a similar internal brace augment via a “box and cross construct” from the patella inferior pole to tibia tuberosity as above before wound closure (Fig. 3).

The patient was placed in bilateral knee brace postoperatively, which was locked in full extension bilaterally for the first 2 weeks. She was regularly reviewed at 2 weekly intervals up to 2 months, increasing her knee flexion progressively by 30° at each review. She was also seen by the physiotherapist immediately and on an outpatient basis, with much of initial therapy focusing on gradual flexion of the knees. Physiotherapy continued up to a year post surgery.

The patient managed to achieve bilateral knee range of motion from 0° to 100° at 3 months post-operatively and her bilateral knee braces were removed thereafter. Her initial progress was conservative as care was taken to ensure adequate soft tissue healing before progression of gait retraining and strengthening. At 5 months, the patient was able to walk without a limp. At 12 months post-operatively, she had good bilateral knee active range of motion of 0° to 130°, with restoration of quadriceps muscle bulk (Fig. 3). She was last reviewed 2.5 years following surgery and has returned to physical activities such as hiking and jogging.

## Discussion

Trampoline-related knee injuries are increasing due to a gain in popularity of its recreational use, with a recent study stating that surgical treatment was required in 28% of injuries, with patella tendon ruptures being one of the less common injuries^1^. In a recently published systematic review on BPT injury, 40 out of the same 45 patients reported either sudden quadriceps contraction or hyperflexion as the mode of injury, which is consistent with the described mechanism in our patient^2^.

Numerous techniques have been described to repair bilateral ruptured patella tendon, and careful selection of appropriate techniques given intra-operative findings are paramount to achieving good outcome^3-4^. In our patient, direct repair was performed for the left knee as the remaining tendon quality was fair and could withstand Krakow technique repair. The patella tendon of the right knee was unsuitable for direct repair hence transosseous repair of the remnant distal patella tendon was performed. In other cases of severely torn patella tendon without remaining substance for repair, the surgical option to consider would be that of patella tendon reconstruction^5^. Regular reviews during the early postoperative period up to six months was crucial to ensure that patient was compliant to knee range of motion precautions and physical therapy instructions to avoid stiffness or failure repair in either knee.

In conclusion, this case report highlights simultaneous bilateral patella tendon rupture from a trampoline injury. Direct repair of the patella tendon can be performed if there is good residual remnant tendon quality and should always be protected with an internal brace augmentation. Through differing surgical techniques and a supervised rehabilitation protocol, this patient went on to have excellent clinical and functional outcomes despite bilateral debilitating knee injury.

## References

[1] Husen M, Engrav SK, Saul D, Stuart MJ, Milbrandt TA, Levy BA (2023). Differences in Trampoline-Related Knee Injuries Between Children and Adults: A Cross-Sectional Study.. Orthop J Sports Med.

[2] Fernandes A, Rufino M, Hamal D, Mousa A, Fossett E, Cheema KS (2023). Simultaneous Bilateral Patellar Tendon Rupture: A Systematic Review.. Cureus.

[3] Biedermann B, Hill W, Karakash WJ, Bolia IK, Rick Hatch GF (2024). Bilateral patellar tendon repair with suture bridge augmentation: A case report.. Trauma Case Rep..

[4] Murphy SM, McAleese T, Elghobashy O, Walsh J (2022). Bilateral patellar tendon rupture following low-energy trauma in a young patient without predisposing risk factors.. Trauma Case Rep.

[5] Husen M, Krych AJ, Poudel K, Stuart MJ (2024). Patellar Tendon Reconstruction After Failed Primary Repair of Bilateral Ruptures: A Case Report.. JBJS Case Connect.

